# Dr. Walker, on Hydrophobia

**Published:** 1808-09

**Authors:** John Walker

**Affiliations:** London


### *To the Editors of the Medical and Physical Journal.*

GENTLEMEN,

THERE is an affecting history of a case of Hydrophobia given by Dr. Bardsley in the Monthly Review for August 1793, from the Memoirs of the Manchester Society, vol. iv. p. 2, on which the Reviewers agree perfectly with the Doctor, that the patient fell a victim to other causes than the poison of a rabid animal. "We have no sufficient evidence," say they, "of canine madness lying dormant for two years; we know that it may arise from accidents very different from the bite of a dog; and this poor patient's sufferings of mind and body cannot fail to excite excite a suspicion, that the cause here did not long precede the effect." John Lindsay, the subject of this case, had been bitten twelve years before. The conclusion of the Author and the Reviewers is most probably correct; yet, among the wonderful resources of Nature in organized bodies, on the occasion of injuries being done them, more particularly in the encystments of extraneous and hurtful substances, whereby they become so completely isolated amid all the currents, streams, distillations, &c. wherewith they are surrounded, as to be out of the reach of the influence of the almost every where acting exhalants and absorbents, these most direct roots and branches of life: even in the changes of parts in the system, such as tubes becoming solid ligaments, when the occasion for their bearing a part in the circulation ceases; and in the occasional indurations of parts which were soft, as in the attachment of the cuticle to the cutis, in cicatrices where the intervening rete mucosum has been destroyed, but which, after a number of years, is sometimes restored, when the little eschar or cicatricle completely disappears. In all these provisions in the animal economy, may we not see the possibility of a particle of the most deleterious matter, getting encysted in the minutest portion of cellular membrane, immediately enveloping it and becoming condensed and indurated around it, while the rest of the saliva of the rabid animal has been completely washed off by the blood and other effused fluid from the wound. If something like this took place in the patient at Manchester, if a particle of the virus lay enveloped in the system for twelve years, which we may perhaps fairly suppose possible if it ever certainly lie dormant for twelve months, then in the excessive sufferings of the poor man, we have perhaps directly physical explanation of the cause of canine madness taking place at the time it did. Almost famished as he must have been at the time, the absorbents avoisining the indurated, the insulated little portion of cellular membrane must have laid hold of it, and broken it down for the support of the general system; the particle of virus then no longer wrapt up in an insusceptible covering, comes in contact with some ramicle of nerve, and the man is lost.

Yours, &c.

JOHN WALKER.

